# Novel application of pluronic lecithin organogels (PLOs) for local delivery of synergistic combination of docetaxel and cisplatin to improve therapeutic efficacy against ovarian cancer

**DOI:** 10.1080/10717544.2018.1440444

**Published:** 2018-02-20

**Authors:** Chia-En Chang, Chien-Ming Hsieh, Ling-Chun Chen, Chia-Yu Su, Der-Zen Liu, Hua-Jing Jhan, Hsiu-O Ho, Ming-Thau Sheu

**Affiliations:** a School of Pharmacy, College of Pharmacy, Taipei Medical University, Taipei, Taiwan, ROC;; b Department of Biotechnology and Pharmaceutical Technology, Yuanpei University of Medical Technology, Hsinchu, Taiwan, ROC;; c Graduate Institute of Biomedical Materials and Engineering, Taipei Medical University, Taipei, Taiwan, ROC;; d Clinical Research Center and Traditional Herbal Medicine Research Center, Taipei Medical University Hospital, Taipei, Taiwan, ROC

**Keywords:** Docetaxel, cisplatin, combination therapy, pluronic lecithin organogel, ovarian cancer

## Abstract

The synergistic combination of docetaxel (DTX) and cisplatin (CIS) by local drug delivery with a pluronic lecithin organogel (PLO) to facilitate high drug concentrations at tumor sites and less nonspecific distribution to normal organs is thought to be beneficial in chemotherapy. In this study, using Capryol-90 (C90) with the addition of lecithin as the oil phase was developed to carry DTX, which was then incorporated into a PLO-containing CIS to formulate a dual-drug injectable PLO for local delivery. An optimal PLO composite, P_13_L_0.15_O_1.5_, composed of PF127:lecithin:C90 at a 13:0.15:1.5 weight ratio was obtained. The sol–gel transition temperature of P_13_L_0.15_O_1.5_ was found to be 33 °C. Tumor inhibition studies illustrated that DTX/CIS-loaded P_13_L_0.15_O_1.5_ could efficiently suppress tumor growth by both intratumoral and peritumoral injections in SKOV-3 xenograft mouse model. Pharmacokinetic studies showed that subcutaneous administration of P_13_L_0.15_O_1.5_ was able to sustain the release of DTX and CIS leading to their slow absorption into the systemic circulation resulting in lower area under the plasma concentration curve at 0–72 h (AUC_0–72_) and maximum concentration (C_max_) values but longer half-life (T_1/2_) and mean residence time (MRT) values. An *in vivo* biodistribution study showed lower DTX and CIS concentrations in organs compared to other treatment groups after IT administration of the dual drug-loaded P_13_L_0.15_O_1.5_. It was concluded that the local co-delivery of DTX and CIS by PLOs may be a promising and effective platform for local anticancer drug delivery with minimal systemic toxicities.

## Introduction

The use of multiple therapeutic agents in combination has become the primary strategy to treat cancer because it suppresses multidrug resistance, reduces individual drug-related toxicity through different mechanisms of action, and generates synergistic anticancer effects (Parhi et al., 2012). Theoretically, combination therapy with therapeutic agents that are demonstrated to be effective as mono-therapy in the clinic should provide better therapeutic effects without additional toxicity. Nevertheless, clinical outcomes of combination therapies are not always as good as anticipated; rather they are often associated with higher toxicities, although non-overlapping toxicity is a major consideration for selecting drug combination candidates (Lee & Nan, 2012). One major factor that separates *in vitro* success from impressive clinical outcomes is low bioavailability, a minimal biodistribution, and inadequate membrane transport properties. Controlling and predicting therapeutic mixtures and providing a better understanding of this increasingly important new paradigm of cancer treatment that reaches diseased tissues and cells has therefore become a major clinical challenge. A common approach is based on tumor biology, molecular pathways, the tumor microenvironment, and tumor–host interactions that are sensitive to both dosing and sequencing of combinatorial drugs. The flourishing strategy to co-deliver drug combinations using a better scheme for precise and controlled delivery has interested an increasing number of researchers since then (Hu & Zhang, 2012).

Several drug carriers, including liposomes (Shaikh et al., 2013; Liu et al., 2014), dendrimers (Cai et al., 2015), silica nanoparticles (Chen et al., 2009), polymeric nanoparticles (Desale et al., 2013), and hydrogels (Cho et al., 2014; Desale et al., 2015; Ma et al., 2015; Wu et al., 2017) have been used to deliver two or more drugs (Hu et al., 2010). CPX-1, a liposome formulation containing irinotecan and floxuridine, is in a phase II trial for treating advanced colorectal cancer (Batist et al., 2008). CPX-351 (VYXEOS™) is another liposome formulation containing cytarabine and daunorubicin developed by Celator^®^ Pharmaceuticals and is in a phase III clinical trial for refractory acute myeloid leukemia (Tardi et al., 2009; Feldman et al., 2011). Another example is Triolimus, a micelle formulation of three drugs (i.e. paclitaxel, rapamycin, and 17-AAG at a 2:1:3 molar ratio) which has been in a phase I clinical trial for angiosarcomas (Hasenstein et al., 2012).

Another approach to co-deliver a combination of two or more drugs is via intratumoral (IT) or peritumoral (PT) injections of drug delivery systems containing drug combinations at a synergistic ratio, which can facilitate drug combinations released at tumor sites with relatively high drug concentrations and less nonspecific distribution of drugs in normal organs (Kim et al., 2010). In this regard, the gel-based system is considered an appropriate formulation for enhancing local drug concentrations at tumor sites and reducing toxic effects in normal cells. Injectable gel systems based on synthetic polymers, proteins, and naturally occurring polysaccharides have been widely used to formulate thermo-reversible gels (Klouda & Mikos, 2008). In particular, injectable *in situ-*forming hydrogels for IT administration (e.g. OncoGel™ and Pluronic^®^ F127 hydrogels) have attracted increasing attention since they provide high regional drug concentrations in tumor sites, sustained release characteristics, low systemic toxicities, minimal invasiveness, biocompatibility, etc. (Elstad & Fowers, 2009; Jhan et al., 2015; Shen et al., 2015).

Pluronic lecithin organogels (PLOs), traditionally applied for the topical and transdermal delivery of drugs, are formed with an appropriate organic solvent or oil (isopropyl myristate or palmitate), phospholipids (lecithin), and a polar solvent containing Pluronic F127 (PF127); these are clear, viscoelastic, thermodynamically stable, biocompatible, and isotropic gels (Avramiotis et al., 2007). PLOs consist of a 3D network of entangled reverse cylindrical micelles, the jelly-like phases, which in many solvents very effectively self-assemble into 3D networks, thereby turning a liquid into a gel. The transitions at the micellar level in a low viscous Newtonian liquid consisting of lecithin reverse micelles in nonpolar organic liquid can contain both water- and oil-soluble ingredients and prolong drug release (Willimann & Luisi, 1991; Kumar & Katare, 2005; Ba et al., 2016). Because of that, PLOs were selected to incorporate dual drugs for local delivery by IT or PT injection.

Docetaxel (DTX) and cisplatin (CIS) are considered the most commonly used antitumor drugs in the clinic against various cancers. DTX is a member of the second-generation taxane class of anticancer agents and is a semisynthetic analog of paclitaxel. It induces apoptotic cell death by cell division cycle arrest at the G_2_/M-phase and tubulin polymerization (Ringel & Horwitz, 1991). In addition, the practical water insolubility of DTX is a major drawback. The action mechanism of CIS is concentration-dependent inhibition of tumor cell growth and DNA synthesis by free radical generation as well as cross-linking to the DNA guanine base (Wang & Lippard, 2005). However, a major limitation of CIS is its indiscriminate distribution in normal tissues, which thereby causes adverse side effects, such as the subsequent discontinuation of CIS therapy that results in poorer therapeutic outcomes, neurotoxicity, myelotoxicity, and nephrotoxicity (DeConti et al., 1973). Therefore, a combination of DTX and CIS was rationally evaluated in this study.

To increase the drug loading (DL) of DTX, Capryol-90 (C90) instead of isopropyl myristate or palmitate was selected as the oil phase, since the maximal solubility of DTX in C90 is as high as 125.86 mg/ml (Yin et al., 2009). In this study, C90 with the addition of lecithin was used as the oil phase to carry DTX, which was then incorporated into the PF127 solution containing CIS to formulate an injectable dual drug-loaded PLO for local delivery. Physical characteristics were examined, including viscoelastic properties, gelation temperature, and drug release of the optimized plain and dual drug-loaded PLOs. The *in vivo* pharmacokinetic profiles, biodistributions, and antitumor efficacies of a synergistic combination of dual drugs loaded in PLOs were assessed in a SKOV-3 xenograft mouse model.

## Materials and methods

### Materials

DTX was supplied by Scino Pharm (Tainan, Taiwan). CIS was purchased from Sigma-Aldrich (St. Louis, MO). PF127 (Kolliphor P407) was obtained from BASF (Ludwigshafen, Germany). Lecithin S100 (soya lecithin) was purchased from Lipoid (Ludwigshafen, Germany). C90 [propylene glycol monocaprylate (type II) NF] was obtained from Gattefosse (Saint Priest, France). All chemicals, solvents, and reagents were of analytical grade. Tynen^®^ as the solvent-based free DTX was purchased from TTY Biopharm (batch no. STW1508, expiration date: 1 February 2018; Taipei, Taiwan).

### Experimental animals

Balb/c nude mice (females, 5 weeks old) and Balb/c mice (females, 5 weeks old) were used for the *in vivo* animal studies. The animals were bought from the National Laboratory Animal Center (Taipei, Taiwan) and maintained in a pathogen-free facility. Animals were housed in groups of five under a 12-h light/dark cycle and allowed food and water *ad libitum*. The animal study was approved by the Laboratory Animal Center of Taipei Medical University (Taipei, Taiwan, approval no. LAC-2014-0244).

### Construction of a sol–gel–sol phase transition diagram for PF127/C90 and evaluation of the sol–gel transition temperature for PF127/lecithin/C90 (PLOs)

The sol–gel–sol transition profiles for PF127/C90 were constructed based on the visual determination of the sol–gel status by a tube inversion method in a 15-mL test tube at a heating rate of 2 °C/10 min (with a temperature scan range of 5–85 °C). The sol–gel or gel–sol transition temperature for the PF127/lecithin/C90 (PLO) system was determined by the flow or non-flow criterion over 1 min with the test tube inverted and a rheometer (for details see ‘Experimental Methods’ section in Supplementary material).

### Rheological characterization of the PF127/lecithin/C90 organogels (PLOs)

All rheological measurements were carried out with a HAAKE Rotational Rheometer RS-1 (DC60/1° Ti) (Thermo Fisher Scientific, Waltham, MA) and a circulating water bath system for temperature control (for details see ‘Experimental Methods’ section in Supplementary material).

### Preparation of a PLO gel incorporating DTX and CIS

DTX and CIS were incorporated into PLOs as follows: DTX was first dissolved in a lecithin/C90 mixture at a weight % ratio of 0.15%/1.5% and allowed to stand overnight at room temperature to ensure complete dissolution. The aqueous phase was prepared by dispersing a weighed amount of PF127 and storage in a refrigerator overnight for full dissolution of PF127 (13%). The next day, CIS was dissolved in the PF127 solution. Finally, the oil phase containing DTX was mixed with the aqueous phase containing CIS at an appropriate volume ratio to give the weight amount of DTX and CIS of 2 mg each, 2 and 1 mg, or 1 and 2 mg in 1 g of the final PLOs under vortexing, which were respectively designated as P_13_L_0.15_O_1.5_^D2C2^, P_13_L_1.5_O_0.15_^D2C1^, and P_13_L_0.15_O_1.5_^D1C2^ as shown by Supplementary Table S2.

### *In vitro* drug release studies

*In vitro* release kinetics of DTX and CIS from P_13_L_0.15_O_1.5_^D2C2^, P_13_L_0.15_O_1.5_^D2C1^, and P_13_L_0.15_O_1.5_^D1C2^ gels were assessed using a dialysis bag method (for details see ‘Experimental Methods’ section in Supplementary material).

### High-performance liquid chromatography-based analysis of DTX and ICP-based analysis of CIS

DTX and CIS concentrations were analyzed by a high-performance liquid chromatography (HPLC) method and Inductively coupled plasma mass spectrometry (ICP-MS) method, respectively (for details see ‘Experimental Methods’ section in Supplementary material).

### *In vitro* synergistic cytotoxicity

A 3-(4,5-dimethylthiazol-2-yl)-2,5-diphenyltetrazolium bromide (MTT) assay was used to measure the cytotoxicity of drug-loaded PLO gels against SKOV-3 ovarian cancer cells (for details see ‘Experimental Methods’ section in Supplementary material).

### *In vivo* tumor inhibition studies

*In vivo* tumor inhibition studies were conducted to assess the efficacy of the IT (directly injected into the tumor) and PT (∼2 mm away from the tumor implantation site) injections. In two separate studies, female nude mice (5 weeks old) bearing human SKOV-3 ovarian cancer cells were randomly divided into eight or six groups (*n* = 5) when the tumor size reached ∼100–200 mm^3^. In order to maintain the same injection volume for every administration, formulations with a concentration at half of the administered dose of both DTX and CIS were prepared for administration. For example, if dosing at 2 mg/kg of each, formulations containing both drugs for administration were prepared at 1 mg/mL of each in water for injection of P_13_L_0.15_O_1.5_, and were designated 2DTX^1^/CIS^1^ and P_13_L_0.15_O_1.5_^2D1C1^, respectively. For IT administration, eight groups were included: the control (PBS), placebo P_13_L_0.15_O_1.5_, 2CIS^2^, 2DTX^2^, 2DTX^1^/CIS^1^, 2DTX^2^/CIS^2^, P_13_L_0.15_O_1.5_^2D1C1^, and P_13_L_0.15_O_1.5_^2D2C2^. Treatments for the IT injection were received three times once every 4 days. Animals were monitored for 33 days and then sacrificed. For PT administration, six groups were included: the control (PBS), placebo P_13_L_0.15_O_1.5_, 2CIS^1^, 2DTX^1^, 2DTX^1^/CIS^1^, and P_13_L_0.15_O_1.5_^2D1C1^. All treatments for PT injections were given three times once every 4 days, and mice were monitored for 42 days and then sacrificed. The dose of various formulation for IT and PT administrations was illustrated in Supplementary Table S3.

Tumor volumes and body weights of animals were monitored at predetermined times. Tumor sizes were measured with electronic calipers, and calculated by the following formula: V = (shortest diameter^2^×longest diameter)/2. The tumor inhibitory rate (R_v_) at the end of the experiment was calculated by the formula: R_v_ = 100%− (V_drug_/V_control_) × 100%, where V_drug_ represents the tumor volume after treatment with the drug, and V_control_ represents the tumor volume after treatment with PBS (Luo et al., 2016).

### Pharmacokinetic study

Balb/c mice were used in the pharmacokinetic studies. Mice were given either intravenous (IV) and subcutaneous (SC) injections of 4 mg/kg of 2DTX^2^ and 2CIS^2^ or a SC injection of 2DTX^1^/CIS^1^, 2DTX^2^/CIS^2^, P_13_L_0.15_O_1.5_^2D1C1^, and P_13_L_0.15_O_1.5_^2D2C2^. The dose of various formulation for IV and SC administrations was illustrated in Supplementary Table S3. Blood was collected at predetermined time points: 0.5, 1, 2, 3, 5, 24, 48, and 72 h. Plasma samples were obtained from centrifuged blood samples at 3500 rpm for 10 min and transported to a freezer at −80 °C until the HPLC and ICP-MS analyzes.

Plasma DTX concentrations were measured with the Waters ACQUITY UPLC-MS/MS system (Waters Corporation, Milford, MA), with a BEH C18 column (1.7 µm, 2.1 × 50 mm). The temperature of the analytical column was set to 40 °C, the flow rate was 0.3 mL/min, and the sample injection volume was 10 µL. The mobile phase was composed of 0.1% formic acid (solvent A) and acetonitrile containing 0.1% formic acid (solvent B) by the gradient mode. The initial condition was 90% solvent A and 10% solvent B for 0.9 min. The buffer ratio was then changed to 0% solvent A and 100% solvent B at 0.9–1.6 min. At the end of 1.6–3.0 min, the buffer ratio was switched to 90% solvent A and 10% solvent B. Positive ion mode electrospray ionization was used for all analyzes (Waters Xevo TQMS, Waters Corporation). The mass spectrometer was operated with a capillary voltage of 3.9 kV, a cone voltage of 65 V, a desolvation temperature of 500 °C, and a desolvation gas flow of 600 L/h.

The procedure for the DTX analysis was as follows. Plasma (20 µL) was mixed with 2 µL of 500 ng/mL paclitaxel, as the internal standard and deproteinized using acetonitrile (75 µL) with vigorous mixing for 10 min. The organic phase was collected after centrifugation at 12,000 rpm for 10 min, and the supernatant was transferred to PhreeTM (Phenomenex^®^). A 10-µL filtered sample was injected into the UPLC-MS/MS system. To assay CIS, plasma samples were analyzed following the same procedures described for assaying plasma samples in the vitro drug release studies.

The related pharmacokinetic parameters including the area under plasma concentration curve (AUC) at 0–72 h (AUC_0–72_), AUC at 0 to infinity (AUC_0–inf_), the time to reach C_max_ (T_max_), maximum concentration of the drug in plasma (C_max_), half-life of the drug (T_1/2_), clearance (CL), volume distribution (V), and the mean residence time (MRT) were analyzed using noncompartmental methods provided by WinNonlin software (v. 6.3.0.395, Pharsight^®^; Princeton, NJ).

### *In vivo* biodistribution studies

Mice with implanted SKOV-3 cells were either given an IV injection of 4 mg/kg of 2DTX^2^ and 2CIS^2^, 2DTX^1^/CIS^1^, 2DTX^2^/CIS^2^, or IT treatment with 2DTX^2^ and 2CIS^2^, 2DTX^1^/CIS^1^, 2DTX^2^/CIS^2^, P_13_L_0.15_O_1.5_^2D1C1^, and P_13_L_0.15_O_1.5_^2D2C2^. The dose of various formulation for IT and PT administrations was illustrated in Supplementary Table S3. At 72 h after treatment, mice were sacrificed, and tissues including the heart, liver, spleen, lungs, kidneys, and tumors were collected after blood perfusion. A five-fold volume of a PBS/0.1% Na_2_EDTA solution was added to each weighed tissue and then homogenized using an SH-100 sample homogenizer (Kurabo Industries, Tokyo, Japan). One hundred microliters of tissue homogenate was collected, and the DTX and CIS concentrations were analyzed following the same procedures described for assaying plasma samples in the pharmacokinetic studies.

### Statistical analysis

All means are presented with their standard deviations (SDs). Differences were considered statistically significant at *p* < .05 using Dunnett’s multi-range test compared to either the PBS control or IV data.

## Results

### Profiling the sol–gel–sol transition temperature of the PF127/C90 systems

In order to develop a thermosensitive hydrogel for the local drug delivery of a synergistic combination of DTX and CIS for chemotherapy, a thermosensitive PF127 hydrogel was incorporated with C90 with the addition of lecithin as a nonpolar phase to formulate PLOs that were presented in the sol state at 4 and 25 °C and transformed to a gel state immediately after the temperature was increased to 37 °C. First, the sol–gel–sol transition diagram was constructed for the PF127 and PF127/C90 systems, and results are shown in Supplementary Figure S1. As illustrated in Supplementary Figure S1, a concentration of PF127 of <15% was unable to form a hydrogel in the PF127 system. However, when C90 was added to the PF127 system at a concentration in the 15–20% range, the sol–gel transition temperature shifted to a temperature lower than room temperature. Therefore, the PF127/C90 system was examined with concentrations of PF127 in the formulations ranging 10.0–16.0% and contents of C90 ranging 1.5–10%, and results are also displayed in Supplementary Figure S1. It demonstrates that at a fixed concentration of PF127, increasing the C90 concentration led to a decrease in the sol-to-gel temperature and an increase in the gel-to-sol temperature. The gelling range between the sol-to-gel temperature and the gel-to-sol temperature became wider with an increasing C90 concentration. However, the dependence of the gelling range on the C90 concentration was diminished with a decrease in the PF127 concentration. It turned out that the sol–gel transition temperature measured by the inversion method for two PF127/C90 gels composed of PF127 at 13 and 14% with the addition of 1.5% C90 appeared to be ∼30 °C. We concluded that the concentration of PF127 in the range of 13–14% with the addition of 1.5% C90 was suitable for the preparation of thermosensitive PF127 hydrogels with a sol–gel transition temperature of 31–33 °C; these gels were in a flowable sol state at 25 °C and became a solid-like gel form at 37 °C.

### Characterization of PLO composed of PF127/C90/lecithin and its optimization

In order to further stabilize PF127/C90 gels, lecithin was added as linker molecules which tended to distribute themselves beside its polar head to the PF127 phase (hydrophilic linkers) and beside its non-polar tail (lipophilic linkers) to the C90 phase. Moreover, combining both hydrophilic and lipophilic linkers improved the solubilization capacity of the system by forming surfactant-like self-assemblies. Based on results revealed above, PLO gels were prepared at two concentrations of PF127 (13 and 14%, w/w) with a mixture of C90 (1.5%) and lecithin in various C90:lecithin ratios of 1:0, 1:0.1, 1:0.2, 1:0.3, 1:0.4, and 1:0.5 (w/w). Supplementary Table S1 reports results of the sol (S) and gel (G) states at 4, 25, and 37 °C for those PLO formulations with various amount of lecithin added as determined by the tube inversion method. The exact sol–gel transition temperature was also measured using a temperature ramp modulus as described in the Supplementary material: ‘Experimental Method’ section and reported in Supplementary Table S1. It demonstrates that only those PLO gels with 13% PF127 and 1.5% C90 possessed a sol–gel transition of >25 °C,  regardless of the concentration of lecithin added. At the same 1.5% C90 concentration, increasing the concentration of lecithin caused the sol-to-gel temperature to only slightly decrease, but it was still above room temperature, and the exact sol–gel temperature was in the range of 28–33 °C. The transparency of PLOs was also found to decrease with an increasing amount of lecithin added (data not shown). PLO gels composed of PF127:lecithin:C90 in a weight % ratio of 13:0.15:1.5 (designated P_13_L_0.15_O_1.5_) were found to be optimal with a sol–gel temperature of 33 °C and a translucent appearance. As a result, PLOs of P_13_L_0.15_O_1.5_ were selected to load dual drugs and were subjected to the following studies.

### Rheological characterization

*In situ* formation of PLOs played an important role in the antitumor effect of IT or PT administration since it determined how soon nanomedicines could be retained in the tumor. *In situ* formation of PLOs was confirmed by examining the tan δ versus the temperature curve. Tan δ (loss factor) is defined as the ratio between the dynamic storage modulus (G′, gel state) and the loss modulus (G′′, sol state) of the viscoelastic deformation behavior. At tan δ < 1 (G′>G′′), a confirmation of the solid state of gels was obtained. On the contrary, at tan δ > 1, the liquid state prevailed. G′, G′′, and tan δ versus temperature profiles for PLO gels are respectively presented in Supplementary Figure S2(A–C). Typically, both G′ and G′′ values for all PLOs examined as shown by Supplementary Figure S2(A,B) slowly or gradually increased with an increasing temperature before reaching the sol–gel transition temperature, during which period G′ was always lower than G′′, indicating that the PLO gels were in a sol state. Sharp increases in both values were observed when the temperature reached the respective sol–gel transition temperature followed by a plateau, at which point, the G′ value was larger than G′′, indicating that the PLOs were in a gel state. As defined by tan δ, Supplementary Figure S2(C) presents the tan δ curve of PLOs examined which were higher than 1 in the low temperature range, indicating a typical sol state. On the other hand, a significant decrease in tan δ was observed with an increasing temperature, indicating a transition to a typical gel state. The sol–gel transition temperature of the PLO gels examined was determined from the intercept point of the line with tan δ = 1 and the tan δ versus temperature curve, and results are listed in Supplementary Table S1. It was apparent that the sol-to-gel transition had a tendency to decrease with a greater amount of lecithin added to C90.

The gel strength of the PLOs determined the retention ability of the *in situ*-formed gel itself and the release rate of the drug from the gel matrix. Therefore, the gel strength of PLOs with the addition of various percentages of lecithin in C90 was evaluated using the storage modulus (G′) of PLOs as an indicator monitored as a function of the amplitude and frequency at 37 °C, at which all PLOs examined were expected to transform into the gel state. The stress range over which G′ is independent of the applied shear stress is the linear viscoelastic region (LVR). Generally, the larger the value of G′ and the longer LVR is, the greater the gel strength is. TLVR profiles of all PLOs were determined by subjecting them to a stress sweep (0.01–100 Pa) until structural breakdown. Supplementary Figure S2(D) shows that those PLO gels with additional lecithin exhibited significantly higher G′ values and longer LVRs compared to those without adding lecithin (P_13_L_0_O_1.5_). This indicates that the addition of lecithin to C90 was able to increase the gel strength of the PLO. Further, frequency sweep profiles in the range of 0.1–10 Hz for PLOs with the addition of various amounts of lecithin are delineated in Supplementary Figure S2(E). It also demonstrates that the G′ of PLOs increased with an increasing shear strain rate in the initial phase and then reached a plateau at a strain rate of ∼2 Hz, followed by a steady G′ value, which slightly increased independently of increases in the added amount of lecithin to the C90 phase compared to that without adding lecithin (P_13_L_0_O_1.5_). It also indicates that the addition of lecithin to PLOs (P_13_L_0.15_O_1.5_) was able to increase the PLO’s gel strength (G′). Both were able to ensure the retention of *in situ*-formed PLOs in the tumor, and the drug was released in a controllable manner when the temperature to which the PLOs were exposed was brought up to body temperature of 37 °C.

Meanwhile, when the PLOs were subjected to an increasing shear rate at 25 °C, at which all PLOs were in a sol state, the viscosity of all PLO samples exhibited a typical shear-thinning character as shown in Supplementary Figure S2(F). This demonstrates that the PLOs displayed a progressive decrease in viscosity to nearly 0, with an increase in the shear rate from 0 to 1000 s^−1^ (Jokerst et al., 2011), which indicates that they were a pseudo-plastic fluid at 25 °C. The lower viscosity in the sol state of the PLOs at low and room temperature ensured that they can easily be injected.

Overall, rheological characterization confirmed that the PLOs developed in this study were thermosensitive hydrogels with a sol–gel temperature of ∼33 °C, lower than that at which PLOs were at a sol state with a shear thinning character that ensures that they can easily be injected at room temperature and higher, than that of *in situ*-formulated PLOs with a high modulus of the gel state at body temperature that would be expected to be beneficial in forming a depot to locally release the synergistic combination of DTX and CIS to effectively inhibit the proliferation of ovarian cancer.

### *In vitro* synergistic cytotoxicity

To verify the synergistic effect of the combination of DTX and CIS co-delivered by PLOs, the *in vitro* antitumor effects of free drugs and drug-loaded PLOs against SKOV-3 cells were examined using an MTT assay. Cell viability effects of the DTX and CIS combination in PLOs possessing various DTX/CIS weight ratios (2/2, 2/1, and 1/2) were evaluated. Results demonstrated that when various ratios of DTX and CIS were co-delivered by P_13_L_0.15_O_1.5_, the cytotoxicity killing ovarian cancer cells was significantly increased compared with free CIS. After 48 h of incubation, all free drugs and drug-loaded PLOs showed dose-dependent cell proliferation-inhibition behavior, and the combination of DTX and CIS led to even greater enhancement of cell proliferation inhibition. It was observed that IC_50_ values of CIS for P_13_L_0.15_O_1.5_^D2C2, D2C1, and D1C2^ were 6.70, 14.50, and 4.41 ng/mL, which were 0.018, 0.29, and 0.04 times lower than the IC_50_ of CIS for those corresponding dose combinations of DTX^2^/CIS^2^, DTX^2^/CIS^1^, and DTX^1^/CIS^2^ (360.93, 50.13, and 117.74 ng/ml), respectively. These data indicate that synergistic cytotoxicity was observed to be profound against SKOV-3 cells using P_13_L_0.15_O_1.5_ as the co-delivery system for DTX and CIS.

To confirm drug synergy in PLOs, we carried out a combination index (CI) determinations. In CI plots (data not shown), P_13_L_0.15_O_1.5_ exhibited the best antitumor activity over a range of drug effect levels (IC_40_–IC_60_) among all drug formulations. CI_50_ values of P_13_L_0.15_O_1.5_^D2C2^, P_13_L_0.15_O_1.5_^D1C2^, and P_13_L_0.15_O_1.5_^D2C1^ were calculated to be 0.073, 0.117, and 0.006, which were respectively 0.084-, 0.003-, and 0.052-times lower than those for corresponding dose combinations of free DTX and free CIS. CI values of P_13_L_0.15_O_1.5_^D2C2^, P_13_L_0.15_O_1.5_^D1C2^, and P_13_L_0.15_O_1.5_^D2C1^ were all <1 and showed an obvious synergistic effect, indicating that the co-delivery of DTX and CIS in P_13_L_0.15_O_1.5_ had evident superiority compared to the free drug combination.

### *In vitro* drug release studies

The drug release behaviors of three PLO formulations (P_13_L_0.15_O_1.5_^D2C2^, P_13_L_0.15_O_1.5_^D2C1^, and P_13_L_0.15_O_1.5_^D1C2^) with different ratios of DTX and CIS combinations were evaluated *in vitro*, and results are presented in Supplementary Figure S3(A,B), respectively. The release profiles of DTX from PLOs at 37 °C indicated a sustained-release manner over 72 h in the release medium. The slow and sustained DTX release from PLO gels might be ascribed to the strong hydrophobic interaction between DTX and the inner core of the PLOs. This drug release behavior suggests that the rate-determining step was the diffusion of DTX through the gel matrix of the PLO. Supplementary Figure S3(B) shows the *in vitro* release profile of CIS, with a rapid and nearly complete extent of release of CIS from all free CIS groups (CIS^2^, DTX^2^/CIS^2^, DTX^2^/CIS^1^, and DTX^1^/CIS^2^) within 3 h in PBS media, whereas the initial release of CIS from PLOs was fast but with less CIS released.

### Tumor inhibition studies

**Figure 1.**
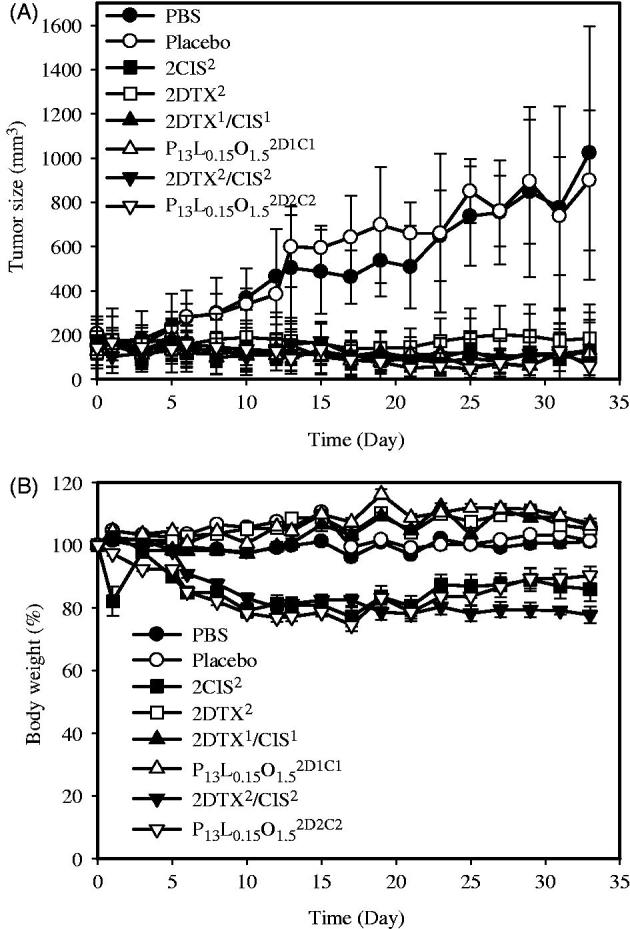
*In vivo* tumor growth inhibition (A) and body weight changes (B) of mice bearing SKOV-3 ovarian cancer xenografts after IT treatment with PBS(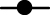), a placebo (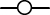), 2CIS^2^ (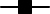), 2DTX^2^ (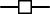), 2DTX^1^/CIS^1^ (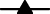), P_13_L_0.15_O_1.5_^2D1C1^ (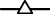), 2DTX^2^/CIS^2^ (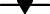), and P_13_L_0.15_O_1.5_^2D1C2^ (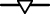) formulations (on Days 0, 4, and 8). Each point is shown as the mean.

The *in vivo* antitumor efficacies of the dual drug-loaded PLOs were evaluated on a nude mice model bearing human ovarian SKOV-3 xenografts by IT and PT injections. Results of the tumor-inhibition profiles and body weight-change profiles are demonstrated in Figure 1(A,B), respectively. Figure 1(A) shows that the tumor inhibitory rate of P_13_L_0.15_O_1.5_^2D2C2^ after an IT injection was 94.22 ± 3.82%, whereas they were 92.84 ± 6.93%, 88.43 ± 12.21%, 87.20 ± 10.51%, 86.69 ± 19.62%, and 81.87 ± 11.51% for 2DTX^2^/CIS^2^, 2CIS^2^, P_13_L_0.15_O_1.5_^2D1C1^, 2DTX^1^/CIS^1^, and 2DTX^2^, respectively. A comparison between treatments with dual agents (2DTX^1^/CIS^1^) (86.69%) and 2CIS^2^ (88.43%) showed a greater inhibition rate than that for 2DTX^2^ (81.87%) at the same dose. This means that CIS was more potent than DTX at inhibiting ovarian cancer proliferation. Given the same dose (4 mg/kg each) of a combination of DTX and CIS, the tumor inhibition rate of 94.22% for P_13_L_0.15_O_1.5_^2D2C2^ was slightly larger than that of 92.84% for 2DTX^2^/CIS^2^ at the end of treatment (Day 33). This indicates that it was beneficial to co-deliver the synergistic combination of DTX and CIS in PLOs. For these treatments given at the same total dose (2CIS^2^, P_13_L_0.15_O_1.5_^2D1C1^, 2DTX^1^/CIS^1^, and 2DTX^2^), the inhibition rate of 87.20% for P_13_L_0.15_O_1.5_^2D1C1^ was slightly lower than that of 88.43% for 2CIS^2^. This implies that the combination of DTX and CIS loaded in PLOs was able to synergistically inhibit tumor proliferation. Further, the inhibition rate of 87.20% for P_13_L_0.15_O_1.5_^2D2C2^ was slightly superior to that of 86.69% for 2DTX^1^/CIS^1^. This was probably due to the drug being loaded in a PLO leading to this synergistic combination of DTX and CIS being maintained. We concluded that PLOs loaded with a synergistic combination of DTX and CIS were able to improve the therapeutic efficacy against ovarian cancer.

Changes in body weight of the treated mice were also monitored throughout the experiment, and it was used as an indication of adverse effects of the anticancer agents. Body weight changes displayed in Figure 1(B) show a greater loss extent (≥20%) of body weight within 10 days for those mice treated with a CIS dose of 4 mg/kg, which included groups treated with 2CIS^2^, 2DTX^2^/CIS^2^, and P_13_L_0.15_O_1.5_^2D2C2^. At the end of the study, however, only the body weight loss of mice in the group treated with 2DTX^2^/CIS^2^ was observed to still be ∼20%. On the contrary, two treatments with CIS at a dose of 2 mg/kg, including 2DTX^1^/CIS^1^ and P_13_L_0.15_O_1.5_^2D1C1^, did not lead to any significant body weight loss, demonstrating that systemic toxicity could be reduced with a reduced dose of CIS and drugs loaded in the PLO. In summary, the dual drug-loaded PLO was able to improve the antitumor efficacy of the synergistic combination of DTX and CIS at a lower dose with reduced drug-related toxicity.

**Figure 2.**
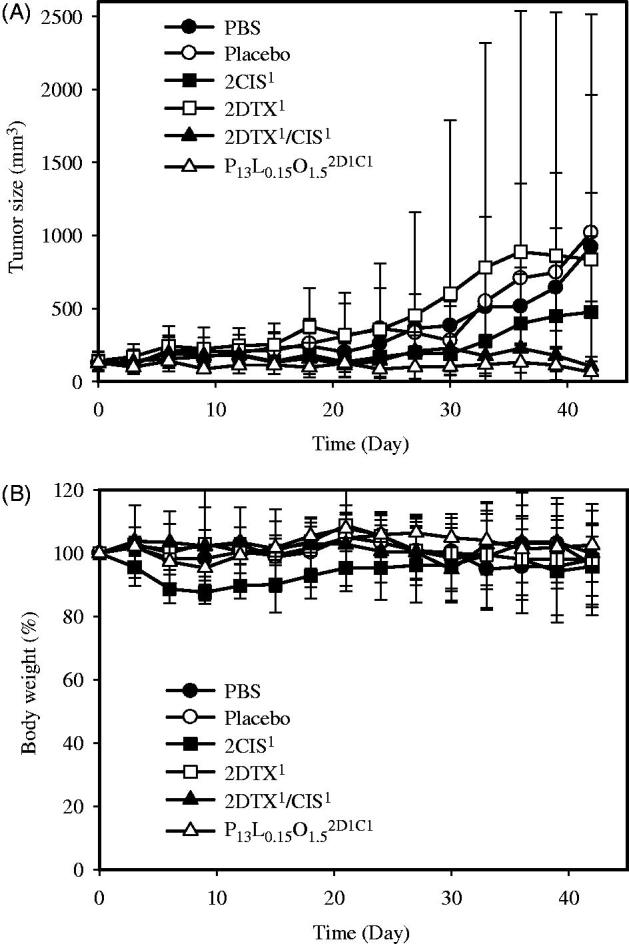
*In vivo* tumor growth inhibition (A) and body weight changes (B) of mice bearing SKOV-3 ovarian cancer xenografts after PT treatment with PBS (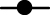), a placebo (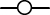), 2CIS^1^ (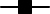), 2DTX^1^ (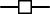), 2DTX^1^/CIS^1^ (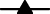), and P_13_L_0.15_O_1.5_^2D1C1^ (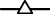) formulations (on Days 0, 4, and 8). Each point is shown as the mean ± SD (*n* = 5).

The *in vivo* antitumor efficacy of a PT injection was also investigated in Balb/c nude mice with SKOV-3 tumor xenografts, and results are illustrated in Figure 2(A,B) for tumor inhibition and body weight change profiles, respectively. In the PBS control group, the average tumor volume on Day 42 was 7.37-times larger than that on Day 0 (Figure 2(A)). Compared to the control group, inhibitory rates of P_13_L_0.15_O_1.5_^2D1C1^, 2DTX^1^/CIS^1^, 2DTX^1^, and 2CIS^1^ over the 42-day period were 93.09 ± 7.90%, 88.71 ± 35.98%, 83.38 ± 19.08%, and 48.36 ± 56.40%, respectively. We observed greater extents of inhibition with P_13_L_0.15_O_1.5_^2D1C1^ and 2DTX^1^/CIS^1^ compared to those with 2DTX^1^ and 2CIS^1^ (93.09 and 88.71% versus 83.38 and 48.36%) at the same total dose amount per kilogram, indicating that the improved therapeutic efficacy was attributed to the synergistic combination of DTX and CIS at a 1:1 ratio. Regarding the weight loss as depicted in Figure 2(B), only the body weights of the 2CIS^1^ treated group showed a slight decrease, while mice in the other groups kept slowly growing or remained unchanged. But one of five mice died on Day 24 due to acute toxicity in the group receiving 2DTX^1^/CIS^1^. None of the mice receiving P_13_L_0.15_O_1.5_^2D1C1^ died during the entire experimental period. These results also suggest that incorporation of a dual drug combination in PLO gels can minimize potential systemic toxicities in addition to improving the efficacy as a result of the synergistic combination compared to that for the free drug combination of 2DTX^1^/CIS^1^. It was concluded that PLO gels loaded with the dual drug combination can enhance the tumor inhibition efficacy and reduce systemic toxicity compared to that for the free drug combination. Further, the study results also implied that even a PT injection was able to exert significant tumor inhibition, and the direct IT injection was not a necessary delivery route for clinical application.

### Pharmacokinetic studies

**Figure 3.**
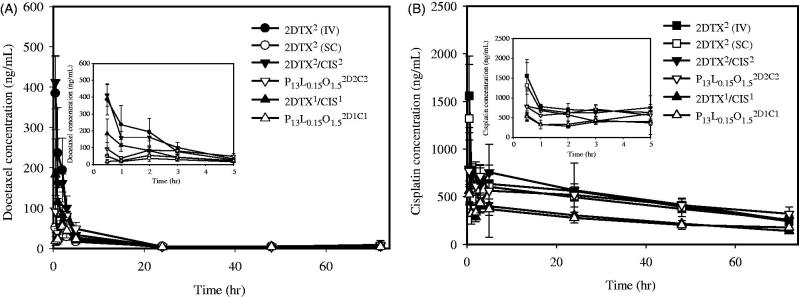
*In vivo* pharmacokinetic profiles of DTX (A) and CIS (B) after being treated with 2DTX^2^ (IV, 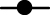), 2CIS^2^ (IV,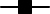) through a tail vein and 2DTX^2^ (SC,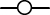), 2CIS^2^ (SC,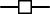), 2DTX^2^/CIS^2^ (SC,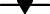), P_13_L_0.15_O_1.5_^2D1C1^ (SC,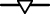), 2DTX^1^/CIS^1^ (SC,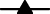), and P_13_L_0.15_O_1.5_^2D1C1^ (SC, 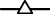). Data are expressed as the mean ± SD (*n* = 3 or 4).

Figure 3 shows the average plasma concentrations versus time curves for DTX (Figure 3(A)) and CIS (Figure 3(B)) after an IV or SC injection of DTX and CIS alone, their combination in solution, and their combination in PLO gels. The related pharmacokinetic parameters we calculated are respectively listed in Tables 1 and 2 for DTX and CIS. As revealed in Table 1, the AUC_0–72_ for DTX by IV administration of 2DTX^2^ was 1387.92 ± 125.25 ng/h/mL, which was 2.37- and 1.25-fold, respectively, higher than that for SC administration of 2DTX^2^/CIS^2^ (1231.37 ± 242.24 ng/h/mL) and P_13_L_0.15_O_1.5_^2D2C2^ (1110.42 ± 181.46 ng/h/mL). Table 1 further reveals that values of the AUC_0–72_ for DTX by SC administration of 2DTX^1^/CIS^1^ and P_13_L_0.15_O_1.5_^2D1C1^ were 816.68 ± 123.29 and 614.65 ± 67.66 ng/h/mL, respectively. C_max_ values for DTX by IV administration of 2DTX^2^ and SC administration of 2DTX^2^/CIS^2^ were 418.33 ± 46.53 and 412.23 ± 66.50 ng/mL, respectively, both of which were significantly higher than that for P_13_L_0.15_O_1.5_^2D2C2^ (108.06 ± 18.43 ng/mL). The C_max_ for DTX by SC administration of P_13_L_0.15_O_1.5_^2D1C1^ was 54.19 ± 6.56 ng/mL, which was an ∼3-fold decrease compared to that for 2DTX^1^/CIS^1^ of 197.74 ± 71.78 ng/mL. Apparently, *in situ* formation of PLO gels after SC administration of P_13_L_0.15_O_1.5_^2D2C2^ and P_13_L_0.15_O_1.5_^2D1C1^ was able to hinder the release of DTX from PLO gels resulting in a lower C_max_ and AUC_0–72_ compared to those for SC administration of the free drug combination. This also indicated that a longer retention of DTX in the injection site was expected.

**Table 1.** Pharmacokinetic parameter estimations of DTX in plasma of normal balb/c mice after being treated with 2DTX^2^ (4 mg/kg, IV) through a tail vein and 2DTX^2^ (4 mg/kg, SC), 2DTX^2^/CIS^2^ (4 mg/kg each, SC), P_13_L_0.15_O_1.5_^2D2C2^ (4 mg/kg each, SC), 2DTX^1^/CIS^1^ (2 mg/kg each, SC), and P_13_L_0.15_O_1.5_^2D1C1^ (2 mg/kg each, SC).

Parameters	2DTX^2^ (IV)	2DTX^2^ (SC)	2DTX^2^/CIS^2^	P_13_L_0.15_O_1.5_^2D2C2^	2DTX^1^/CIS^1^	P_13_L_0.15_O_1.5_^2D1C1^
AUC_0–72_ (ng/h/mL)	1387.92 (125.25)	584.56 (34.51)	1231.37 (242.24)	1110.42 (181.46)	816.68 (123.29)	641.65 (67.66)
AUC_0–inf_ (ng/h/mL)	1454.96 (116.97)	820.32 (158.14)	1353.92 (366.51)	1489.74 (724.61)	1094.81 (512.935)	787.47 (85.82)
*C*_max_ (ng/mL)	418.33 (46.53)	52.76 (10.98)	412.23 (66.50)	108.06 (18.43)	197.74 (71.78)	54.19 (6.56)
*T*_max_ (h)	0.63 (0.25)	0.88 (0.75)	0.5 (0)	0.88 (0.75)	0.75 (0.29)	2.0 (0.00)
*T*_1/2_ (h)	11.86 (1.27)	28.56 (4.83)	14.45 (2.48)	19.57 (10.38)	18.97 (6.60)	22.52 (3.16)
CL (mL/h/kg)	2762.58 (221.83)	4998.16 (848.12)	3089.60 (667.77)	3102.56 (1180.29)	2079.57 (739.80)	2560.50 (253.71)
*V* (mL/kg)	47,222.73 (5646.61)	201,497.3 (4984.60)	64,173.62 (18,284.55)	75,234.89 (5935.48)	52,925.09 (15,376.22)	82,359.13 (3530.80)
MRT (h)	8.50 (1.69)	23.92 (1.80)	10.87 (1.94)	16.36 (6.78)	18.48 (9.12)	19.37 (3.48)

Data are presented as the mean (SD) (*n* = 3 or 4).

AUC_0-72_: area under the receiver operating curve at 0–72 h; AUC_0–inf_: area under the receiver operating curve at 0 to infinity; *C*_max_: maximum plasma drug concentration; *T*_max_: the time to reach C_max_; *T*_1/2_: half-life of the drug; CL: clearance; V: volume of the distribution; MRT: mean residence time; IV: intravenous; SC: subcutaneous.

**Table 2.** Pharmacokinetic parameter estimations of CIS in plasma of normal balb/c mice after being treated with 2CIS^2^ (4 mg/kg, IV) through a tail vein and 2CIS^2^ (4 mg/kg, SC), 2DTX^2^/CIS^2^ (4 mg/kg each, SC), P_13_L_0.15_O_1.5_^2D2C2^ (4 mg/kg each, SC), 2DTX^1^/CIS^1^ (2 mg/kg each, SC), and P_13_L_0.15_O_1.5_^2D1C1^ (2 mg/kg each, SC).

Parameters	2CIS^2^ (IV)	2CIS^2^ (SC)	2DTX^2^/CIS^2^	P_13_L_0.15_O_1.5_^2D2C2^	2DTX^1^/CIS^1^	P_13_L_0.15_O_1.5_^2D1C1^
AUC_0–72_ (ng/h/mL)	35,030.66 (3817.203)	31,398.67 (1941.69)	35,684.62 (2738.70)	33,273.03 (6790.87)	19,090.21 (1003.34)	18,396.05 (3127.88)
AUC_0–inf_ (ng/h/mL)	48,701.88 (7414.28)	65,844.12 (40,991.26)	51,110.11 (469.51)	65,781.19 (5658.28)	27,883.53 (2270.33)	36,781.17 (15,477.15)
C_max_ (ng/mL)	1558.51 (330.03)	1320.87 (655.35)	932.62 (250.07)	906.87 (92.68)	561.59 (45.50)	531.64 (91.11)
T_max_ (h)	0.5 (0.00)	0.5 (0.00)	2.17 (2.46)	2.00 (2.60)	0.5 (0.00)	0.67 (0.29)
T_1/2_ (h)	37.63 (12.40)	75.24 (61.61)	42.12 (7.25)	70.06 (9.46)	42.97 (9.78)	66.83 (27.03)
CL (mL/h/kg)	83.32 (11.68)	76.34 (38.80)	78.27 (0.722)	39.57 (34.51)	72.04 (5.70)	60.11 (20.48)
V (mL/kg)	4440.05 (1328.08)	6123.76 (1212.61)	4751.30 (779.98)	4235.26 (3810.24)	4416.04 (658.35)	5303.11 (945.78)
MRT (h)	28.26 (1.27)	29.99 (1.74)	29.02 (0.63)	21.16 (16.18)	28.97 (0.85)	30.00 (1.99)

Parameters are defined in the footnotes to Table 1.

As revealed by Table 2, the AUC_0–72_ for CIS by IV administration of 2CIS^2^ was 35,030.66 ± 3817.20 ng/h/mL, which was 1.12-, 0.98-, and 1.05-fold, respectively, higher than those for the SC administration of 2CIS^2^ (3,1398.67 ± 1941.69 ng/h/mL), 2DTX^2^/CIS^2^ (35,684.62 ± 2738.70 ng/h/mL), and P_13_L_0.15_O_1.5_^2D2C2^ (33,273.03 ± 6790.87 ng/h/mL). The C_max_ for CIS by IV administration of 2CIS^2^ (1558.51 ± 330.03 ng/mL) was 1.18-, 1.67- and 1.72-fold higher than those for the SC administration of 2CIS^2^ (1320.87 ± 655.35 ng/mL), 2DTX^2^/CIS^2^ (932.62 ± 250.07 ng/mL), and P_13_L_0.15_O_1.5_^2D2C2^ (906.87 ± 92.68 ng/mL), respectively. Table 2 further reveals that values of the AUC_0–72_ for CIS by SC administration of 2DTX^1^/CIS^1^ and P_13_L_0.15_O_1.5_^2D1C1^ were 19,090.21 ± 1003.34 and 18,396.05 ± 3127.88 ng/h/mL, respectively. The C_max_ for CIS by SC administration of P_13_L_0.15_O_1.5_^2D1C1^ was 531.64 ± 91.11 ng/mL, which was an ∼1.06-fold decrease compared to that for 2DTX^1^/CIS^1^ at 561.59 ± 45.50 ng/mL. The results suggest that SC administration of PLO gels containing the synergistic combination of DTX and CIS was able to delay the absorption of CIS into the systemic circulation, resulting in lower C_max_ and AUC values.

### *In vivo* biodistribution studies

**Figure 4.**
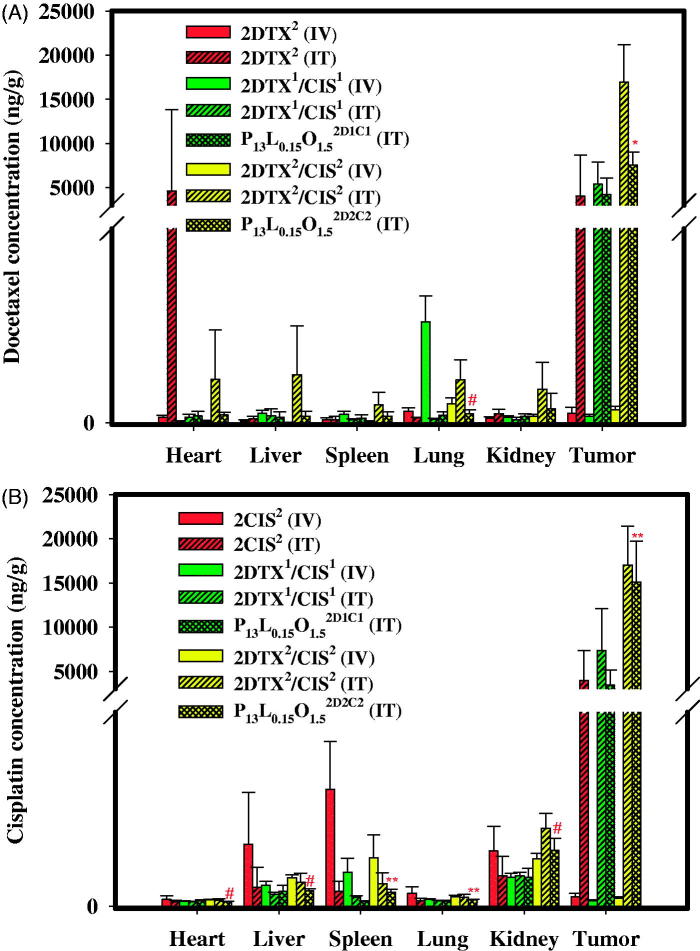
Tissue distributions of DTX (A) and CIS (B) 72 h after IV and IT administration of 2DTX^2^ (4 mg/kg, IV), 2CIS^2^ (4 mg/kg, IV), 2DTX^2^ (4 mg/kg, IT), 2CIS^2^ (4 mg/kg, IT), 2DTX^1^/CIS^1^ (2 mg/kg each, IV), 2DTX^1^/CIS^1^ (2 mg/kg each, IT), P_13_L_0.15_O_1.5_^2D1C1^ (2 mg/kg each, IT), 2DTX^2^/CIS^2^ (4 mg/kg each, IV), 2DTX^2^/CIS^2^ (4 mg/kg each, IT), and P_13_L_0.15_O_1.5_^2D2C2^ (4 mg/kg each, IT) into SKOV-3 tumor-bearing Balb/c nude mice. Data are expressed as the mean ± SD (*n* = 4).

Biodistributions of these two drugs in various tissues and organs were assessed in nude mice with SKOV-3 implantation after IV or IT administration of 2DTX^2^ (4 mg/kg), 2CIS^2^ (4mg/kg), 2DTX^1^/CIS^1^ (2 mg/kg each), and 2DTX^2^/CIS^2^ (4 mg/kg each) and IT administration of P_13_L_0.15_O_1.5_^2D1C1^ (2 mg/kg each), and P_13_L_0.15_O_1.5_^2D2C2^ (4 mg/kg each). At 72 h after treatment, mice were sacrificed, and tissues including the heart, liver, lungs, spleen, kidneys, and tumors were taken. The biodistribution data of DTX and CIS in these tissues and tumors are presented in Figure 4(A,B), respectively. As shown in Figure 4(A), DTX accumulation values after IT administration of P_13_L_0.15_O_1.5_^2D2C2^ in the heart, liver, spleen, lung, and kidney were 0.18, 0.13, 0.26, 0.21, and 0.42-fold lower, respectively, than those for 2DTX^2^/CIS^2^. More importantly, drug retention in the tumor after IT administration of P_13_L_0.15_O_1.5_^2D2C2^ exhibited a higher level than that of 2DTX^2^ (4 mg/kg, IV) (*p* < .001), and the peak concentration of P_13_L_0.15_O_1.5_^2D2C2^ in tumors was 75,279.33 ± 14824.63 ng/g, representing a 76.92-fold increase over 2DTX^2^ (4 mg/kg, IV). Figure 4(B) illustrates that the distributions of CIS in tissues including the heart, liver, spleen, lungs, and kidneys were at a smaller or comparable amount when P_13_L_0.15_O_1.5_^2D2C2^ was administered through the IT route compared to those of 2DTX^2^/CIS^2^ given by the same route. Furthermore, CIS and DTX concentrations in tumors for both the P_13_L_0.15_O_1.5_^2D1C1^ and P_13_L_0.15_O_1.5_^2D2C2^ groups were higher than those for the 2DTX^2^/CIS^2^ group given by IV administration. The decreased kidney uptake and increased tumor retention suggest increased efficacy and reduced renal toxicity for both the P_13_L_0.15_O_1.5_^2D1C1^ and P_13_L_0.15_O_1.5_^2D2C2^ groups. The improved efficacy of tumor inhibition by P_13_L_0.15_O_1.5_ might be attributed to a greater extent of drug retention of DTX and CIS at a synergistic combination ratio within the tumor.

## Discussion

Common chemotherapy is often associated with adverse effects on normal cells and tissues. Drug delivery systems derived from liposomes, dendrimers, polymeric nanoparticles, and micelles are currently under preclinical and clinical development as novel nanomedicines that can deliver combinations of multiple drugs to various cancers while minimizing systemic toxicities. As an alternative approach, localized chemotherapy can provide clinical benefits due to sustained release of chemotherapeutics at the tumor site with a decreasing extent of systemic toxicity. Therefore, thermosensitive hydrogels as drug delivery systems for chemotherapeutics have garnered increasing attention. The thermosensitive gel is a free-flowing liquid at room temperature and instantly forms a gel depot upon injection into a body site at 37 °C. Herein, PLOs with thermosensitive properties to locally deliver a synergistic combination of dual anticancer drugs were optimally developed for a novel application in chemotherapy. With substitution of traditionally used isopropyl myristate or palmitate in PLOs with C90 to enhance the solubility of hydrophobic cancer drugs, the PLO (P_13_L_0.15_O_1.5_) composed of PF127, lecithin, and C90 at a 13:0.15:1.5 ratio was optimized in this study with characteristics of being a transparent liquid at 4 and 25 °C, and it transforms to a gel in <1 min after the temperature has increased to 37 °C (Supplementary Figure S2(C)). Further, the syringe-ability of P_13_L_0.15_O_1.5_ can be attributed to the sol state character at 25 °C and the shear-thinning behavior with increasing shear stress encountered during injection. The low viscosity of P_13_L_0.15_O_1.5_ ensures that it can easily be injected at low temperatures, while the higher gel strength of PLO gels transformed at body temperature is beneficial for forming a depot to control drug release. Shear thinning means that the viscosity of P_13_L_0.15_O_1.5_ had accordingly decreased when the shear rate increased during the injection, further facilitating the syringe-ability of P_13_L_0.15_O_1.5_ (Supplementary Figure S2(F)).

DTX is hydrophobic and practically insoluble in water (4.93 µg/mL) (Gao et al., 2008). Thus, C90 was chosen to replace isopropyl myristate or palmitate as the oil phase in this study to increase its DL in PLO. According to the proposed model by Ruiz et al. (2007), when C90/lecithin was added to the PF127 solution, the surfactant micelles formed by PF127 were grouped around the phospholipid hydrophobic tail groups to form vesicles with a hydrophilic surface potentially able to carry active water-soluble principles, surrounding a hydrophobic compartment potentially able to carry active liposoluble principles. These PLO unilamellar water/oil vesicles were similar to those in other carrier systems such as liposomes.

Longer retention of a drug in tumor tissues with sustained release is beneficial for cancer chemotherapy to improve the antitumor efficacy and alleviate severe side effects (Cheng et al., 2013; Wu et al., 2014; Fan et al., 2015). Sustained drug release, which is considered a necessary prerequisite for localized tumor therapy, was observed over 3 days from P_13_L_0.15_O_1.5_^D2C2^ at 37 °C. Typically, only 33% of the accumulative DTX amount was released from P_13_L_0.15_O_1.5_^D2C2^ within 12 h, whereas 65 and 59% of DTX were released from DTX and DTX^2^/CIS^2^, respectively. However, CIS release profiles from PLO gels at pH 7.4 exhibited a burst release. A fast release of CIS from all formulations might be attributed to a higher solubility of CIS in the release medium, while the smaller extent of CIS released from PLOs might be explained by the fact that CIS is possibly absorbed onto the PLO matrix (Cho et al., 2009).

In this study, *in vitro* synergistic cytotoxicity was performed to evaluate the inhibitory effect of DTX plus CIS on the growth of SKOV-3 cells. CI_50_ values of DTX and CIS in free form were larger than those incorporated in PLOs. The results indicate that P_13_L_0.15_O_1.5_ can deliver a synergistic combination of DTX and CIS to enhance the cytotoxicity against SKOV-3 cells.

The inhibitory effects on tumor proliferation in nude mice with SKOV-3 xenografts demonstrated that the control group (PBS) and P_13_L_0.15_O_1.5_ placebo showed no substantial inhibitory effects on SKOV-3 tumor growth, and the average tumor volume rapidly increased to >1000 mm^3^ at 33 days after IT administration. Compared to the PBS control group, the tumor inhibitory rate on Day 33 after IT administration with P_13_L_0.15_O_1.5_^2D2C2^ was 94.22%, followed by 92.84% for 2DTX^2^/CIS^2^, 88.43% for 2CIS^2^, 87.20% for P_13_L_0.15_O_1.5_^2D1C1^, 86.69% for 2DTX^1^/CIS^1^, and 81.87% for 2DTX^2^. For PT administration, inhibitory rates of P_13_L_0.15_O_1.5_^2D1C1^, 2DTX^1^/CIS^1^, 2DTX^1^, and 2CIS^1^ were 93.09, 88.71, 83.48, and 48.36%, respectively.

Taken together with the results of the *in vitro* cytotoxicity experiments and *in vivo* antitumor study, the enhanced antitumor activity can be explained by the following aspects: an *in situ*-forming gel by an IT injection rendered continuous release of DTX and CIS within the tumor mass, thus inducing a long-lasting effect of tumor growth inhibition. The co-delivery of DTX and CIS was more effective than the use of the single drugs. For the same drugs, P_13_L_0.15_O_1.5_^2D1C1^ showed a slight better antitumor effect compared to the free drug combination (2DTX^1^/CIS^1^), and similar results were observed with the drug combination. The highest antitumor activity was observed in the P_13_L_0.15_O_1.5_^2D1C1^-treated group, with almost complete inhibition of tumor growth and no obvious tumor recrudescence during the entire treatment.

For the IT administration study, it was expected that mice treated with either 2CIS^2^, 2DTX^2^/CIS^2^, or P_13_L_0.15_O_1.5_^2D2C2^ would display obvious body weight loss. However, in the 2CIS^2^ and P_13_L_0.15_O_1.5_^2D2C2^ treatment groups, an initial weight loss was observed followed by recovery of the body weight after Days 11 and 17, respectively, but the body weight loss of mice in the 2DTX^2^/CIS^2^ group still exceeded 20% on Day 19, suggesting severe systemic toxicity. For the PT administration study, mice treated with 2CIS^1^ displayed slight body weight loss, suggesting some extent of systemic toxicity. Nevertheless, when DTX and CIS were loaded in P_13_L_0.15_O_1.5_^2D1C1^, no difference in body weight was observed compared to the control group (PBS) and placebo P_13_L_0.15_O_1.5_. Although the tumor inhibitory rate between DTX/CIS-loaded PLOs and free formulations was not statistically different, the less decrease of body weight indicates that the carrier had no systemic toxicity and also could efficiently mitigate the systemic toxicity of the incorporated free drugs.

Undesirable side effects due to the absorption of drugs from the injection site into the systemic circulation were reduced because anticancer drugs could be localized in specific sites. In fact, depot systems, including thermoresponsive hydrogels, were shown to be advantageous over conventional free drugs because local concentrations at specific sites can be significantly elevated. In addition, systemic circulation of DTX and CIS could be suppressed, because DTX and CIS were slowly released from P_13_L_0.15_O_1.5_ as revealed with *in vitro* drug release. Thus, PLOs can be promising *in situ-*gelation depot systems aimed at prolonging the release of DTX and CIS from tumor sites for an extended period.

According to the pharmacokinetics studies, AUC_0–72_ and C_max_ values of DTX after SC administration of P_13_L_0.15_O_1.5_^2D2C2^ were 0.902- and 0.262-fold lower than those after SC administration of 2DTX^2^/CIS^2^, respectively. Moreover, the expected lower AUC_0–72_ values for P_13_L_0.15_O_1.5_^2D2C2^ and P_13_L_0.15_O_1.5_^2D1C1^ treatment compared to the corresponding 2DTX^2^/CIS^2^ and 2DTX^1^/CIS^1^ demonstrated that the absorption of DTX and CIS into the bloodstream after being incorporated into a PLO gel was reduced. T_1/2_ and MRT values of DTX after SC administration of P_13_L_0.15_O_1.5_^2D2C2^ were 1.354- and 2.413-fold longer than those for 2DTX^2^/CIS^2^, respectively. The T_1/2_ value of CIS after SC administration of P_13_L_0.15_O_1.5_^2D2C2^ was 1.663-fold longer than that for 2DTX^2^/CIS^2^. These data indicate that P_13_L_0.15_O_1.5_ was able to sustain the release of DTX and CIS as a rate-determining step in the process of drug absorption into the systemic circulation resulting in lower AUC_0–72_ and C_max_ values but longer T_1/2_ and MRT values.

In biodistribution studies after IT administration of P_13_L_0.15_O_1.5_^2D2C2^, it was also noted that the accumulation of DTX and CIS in the heart, liver, spleen, lungs, and kidneys were reduced compared to the group treated with 2DTX^2^/CIS^2^. As evidenced from these biodistribution values, it could be concluded that P_13_L_0.15_O_1.5_ used to co-deliver DTX and CIS at a synergistic combination for treating ovarian cancer was able to reduce toxicity to other tissues and improve the therapeutic index.

## Conclusions

We present here a PLO with replacement of isopropyl myristate or palmitate by C90 as a strategy to co-deliver DTX and CIS at a synergistic ratio to enhance the chemotherapeutic efficacy. The resultant P_13_L_0.15_O_1.5_ containing DTX and CIS at three different ratios showed some synergistic effects in inhibiting the proliferation of SKOV-3 human ovarian cancer cells. An *in vivo* study demonstrated that P_13_L_0.15_O_1.5_ loaded with dual drugs at a 1:1 ratio exhibited enhanced inhibition against SKOV-3 tumor growth compared to that for the single anticancer agents (DTX and CIS) or dual-anticancer drugs (DTX/CIS). Most importantly, P_13_L_0.15_O_1.5_ was able to retain the anticancer drugs at the local injection site of the tumor resulting in enhancement of the therapeutic efficacy with minimization of side effects due to a smaller fraction of the anticancer drug accumulating in normal organs. Therefore, it was concluded that P_13_L_0.15_O_1.5_ is a potential dual-drug co-delivery system for local delivery with a synergistic effect on the chemotherapeutic efficacy.

## Supplementary Material

IDRD_Sheu_et_al_Supplemental_Content.docx
