# Supplementary material for: Novel application of pluronic lecithin organogels (PLOs) for local delivery of synergistic combination of docetaxel and cisplatin to improve therapeutic efficacy against ovarian cancer
Source: Drug Deliv. 2018 Feb 20;25(1):632–43. doi: 10.1080/10717544.2018.1440444 (PMC6058476; doi:10.1080/10717544.2018.1440444)
Supplement: IDRD_Sheu_et_al_Supplemental_Content.docx [file IDRD_A_1440444_SM6077.docx]

**Supporting information**

**Experimental Methods**

**Construction of a sol-gel-sol phase transition diagram for PF127/C90 and evaluation of the sol-gel transition temperature for PF127/lecithin/C90 (PLOs)**

In order to develop a thermosensitive hydrogel based on PF127 with the ability to solubilize a hydrophobic anticancer drug, C90 that is able to solubilize a greater extent of DTX was selected for incorporation into the PF127 hydrogel with/without the addition of lecithin. After that, sol-gel-sol transition profiles for PF127/C90 were constructed with respect to their concentrations. PF127 was prepared simply by adding PF127 to ice-cold water and placing it in a refrigerator to ensure that it completely dissolved, and then the PF127/C90 system was prepared by mixing the oil phase of C90 and the aqueous phase of the PF127 solution. PF127 and C90 were comprised in the ranges of 10.0%~20.0% and 0%~10.0%, respectively. The sol-gel or gel-sol phase transition was visually determined by a tube inversion method in a 15-mL test tube at a heating rate of 2 °C/10 min (with a temperature scan range of 5~85 °C). To evaluate the sol-gel transition temperature for the PF127/lecithin/C90 (PLO) system, the aqueous phase of the PF127 solution at two fixed weight percentages of 13% and 14% was mixed under vortexing with C90 as the oil phase containing lecithin at various ratios of 1:0, 1:0.1, 1:0.2, 1:0.3, 1:0.4, and 1:0.5 (w/w) as listed in Table S1. The oil phase was prepared by mixing C90 and lecithin overnight to ensure that they completely dissolved. The sol-gel or gel-sol transition temperature was determined by the flow or non-flow criterion over 1 min with the test tube inverted and a rheometer.

**Rheological characterization of the** **PF127/lecithin/C90 organogels (PLOs)**

All rheological measurements were carried out with a HAAKE Rotational Rheometer RS-1 (DC60/1^o^ Ti) (Thermo Fisher Scientific, Waltham, MA, USA) and a circulating water bath system for temperature control. A temperature ramp was used to investigate the thermal behavior of the PLOs, including confirmation of the exact sol-gel transition temperature for PLOs. To ensure that the experimental conditions did not interfere with the gelation process, measurements were taken at a low shear stress (1 Pa) and constant frequency (1 Hz) at a heating rate of 1.5 °C/min. The mechanical strength properties of the PLOs were assessed by oscillation amplitude and oscillation frequency sweeps, and the resulting G’ values were monitored at 37 °C, at which PLOs were expected to transform into a gel state. The oscillation amplitude sweep was performed in a shear stress range of 0.01~100 Pa at a constant frequency of 1 Hz. The oscillation frequency sweep was performed in a frequency range of 0.1~10 Hz at a constant shear stress of 1 Hz. Since PLOs were expected to be in a sol state at 25 °C, the shear thinning behavior of the samples was characterized over a range of 0~1000 s^−1^ for a period of 240 s, and the resulting viscosity profiles were recorded.

**In vitro drug release studies**

In vitro release kinetics of DTX and CIS from P_13_L_0.15_O_1.5_^D2C2^, P_13_L_0.15_O_1.5_^D2C1^, and P_13_L_0.15_O_1.5_^D1C2^ gels were assessed using a dialysis bag method. Briefly, 100 µl of a sample was added to a dialysis bag (with a molecular weight cutoff of 6000 Da) and then incubated in 20 mL of phosphate-buffered saline (PBS; pH 7.4) containing 0.5% Tween 80 with mild shaking at 100 rpm and 37 °C. At specified intervals, all releasing medium was withdrawn and replaced with an equal volume of fresh medium. Amounts of DTX and CIS in the collected medium were analyzed by a high-performance liquid chromatography (HPLC) method and inductively coupled plasma mass spectrometry (ICP-MS), respectively, both of which are described below.

**HPLC-based analysis of DTX and ICP-based analysis of CIS**

DTX concentrations were analyzed by an HPLC method, and the sample solution was injected into an Inertsil 6 ODS-3 column (6 µm, 150 × 4.6 mm; GL Sciences, Torrance, CA, USA). The column oven temperature was set to 40 °C. The mobile phase consisted of acetonitrile and water (600:400, volume ratio). The UV detector was set to 227 nm, and the sample injection volume was 20 µL with a flow rate of 1 mL/min. ICP-MS (X series II, Thermo Scientific, Waltham, MA, USA) was used for the quantitative determination of platinum (Pt) levels based on the most abundant isotopes of Pt at m/z 195. Samples were digested in a 3-fold volume of concentrated HNO_3_ for 90 min at 90 °C. The digested sample was further diluted with deionized water to volume bringing the final HNO_3_ concentration to 3.5% (v/v), and samples were stored at 4 °C until Pt was analyzed.

**In vitro synergistic cytotoxicity**

To measure the cytotoxicity of drug-loaded PLO gels, we used a 3-(4,5-dimethylthiazol-2-yl)-2,5-diphenyltetrazolium bromide (MTT) assay against SKOV-3 ovarian cancer cells. SKOV-3 ovarian cancer cells were seeded into 24-well plates at a density of 5×10^4^ cells/well which were grown in Dulbecco’s modified Eagle’s medium (DMEM) with L-glutamine containing 10% fetal bovine serum, supplemented with 50 U/mL penicillin and 50 U/mL streptomycin, and incubated at 37 °Cin a 5% CO_2_ atmosphere. After 24 h of culture, various concentrations of the drug-loaded PLO gel were added to the cell medium and then incubated for 48 h, after which the MTT reagent was added to each well. The optical density (OD) was determined by measuring the absorbance at 570 nm using a plate reader and calculated by the following formula [(OD_treat_ - OD_blank_) / (OD_control_ - OD_blank_) × 100%] of triplicate wells. The effective method to evaluate synergistic drug combinations in vitro is a median-effect analysis, as originally proposed by Chou and Talalay (Chou and Talalay, 1984). The combination index (CI) was used to evaluate synergy between DTX and CIS against SKOV-3 cells in vitro and calculated according to their equation for a two-drug combination analysis:

$CIx=\frac{D}{\mathrm{Dx}}+\frac{C}{\mathrm{Cx}}$;

where D and C denote IC_x_ values of DTX and CIS in combination therapy that kill x% of cell. D_x_ and C_x_ denote doses of DTX and CIS that kill x% cells alone, respectively.

Values of CI = 1, CI < 1, and CI > 1 respectively indicate additivity, synergy, and antagonism.

**Table S1.** Effect of the Pluronic F127/lecithin/C90 content on the status of PLO gels at 4, 25, and 37 °C and their sol-gel transition temperature.

| **Formulation** | **PF127 (%)** | **Lecithin**  **(%)** | **C90**  **(%)** | **4 °C** | **25 °C** | **37 °C** | **Gel Temp.**  **(°C)^a^** |
| --- | --- | --- | --- | --- | --- | --- | --- |
| P_13_L_0_O_1.5_ | 13 | 0 | 1.5 | S | S | G | 33 |
| P_14_L_0_O_1.5_ | 14 | 0 | 1.5 | S | G | G | 19 |
| P_13_L_0.15_O_1.5_ | 13 | 0.15 | 1.5 | S | S | G | 33 |
| P_14_L_0.15_O_1.5_ | 14 | 0.15 | 1.5 | S | G | G | 23 |
| P_13_L_0.3_O_1.5_ | 13 | 0.30 | 1.5 | S | S | G | 28 |
| P_14_L_0.3_O_1.5_ | 14 | 0.30 | 1.5 | S | G | G | 20 |
| P_13_L_0.45_O_1.5_ | 13 | 0.45 | 1.5 | S | S | G | 28 |
| P_14_L_0.45_O_1.5_ | 14 | 0.45 | 1.5 | S | G | G | 17 |
| P_13_L_0.6_O_1.5_ | 13 | 0.60 | 1.5 | S | S | G | 30 |
| P_14_L_0.6_O_1.5_ | 14 | 0.60 | 1.5 | S | G | G | 19 |
| P_13_L_0.75_O_1.5_ | 13 | 0.75 | 1.5 | S | S | G | 30 |
| P_14_L_0.75_O_1.5_ | 14 | 0.75 | 1.5 | S | G | G | 19 |

^a^ Measured by a rheometer.

PF127, Pluronic F127; C90, Capryol 90; Gel Temp, Gel temperature.**Table S2.** PLO gel incorporating different docetaxel and cisplatin.

| Formulation | PF127  % | Lecithin % | C90  (%) | Docetaxel (mg/g) | Cisplatin (mg/g) |
| --- | --- | --- | --- | --- | --- |
| P_13_L_0.15_O_1.5_^D2C2^ | 13 | 0.15 | 1.5 | 2 | 2 |
| P_13_L_0.15_O_1.5_^D1C2^ | 13 | 0.15 | 1.5 | 2 | 1 |
| P_13_L_0.15_O_1.5_^D1C2^ | 13 | 0.15 | 1.5 | 1 | 2 |

PF127, Pluronic F127; C90, Capryol 90

**Table S3.** The dose of various formulations for in vivo tumor inhibition, pharmacokinetic and biodistribution studies.

| Formulations | Docetaxel (mg/kg) | Cisplatin (mg/kg) |
| --- | --- | --- |
| PBS | 0 | 0 |
| Placebo P_13_L_0.15_O_1.5_ | 0 | 0 |
| 2CIS^2^ | 0 | 4 |
| 2DTX^2^ | 4 | 0 |
| 2CIS^1^ | 0 | 2 |
| 2DTX^1^ | 2 | 0 |
| 2DTX^1^/CIS^1^ | 2 | 2 |
| 2DTX^2^/CIS^2^ | 4 | 4 |
| P_13_L_0.15_O_1.5_^2D1C1^ | 2 | 2 |
| P_13_L_0.15_O_1.5_^2D2C2^ | 4 | 4 |

**Figure S1.** Sol-gel-sol phase transition diagram of Pluronic F127/Capryol 90 gel composed of 10-20% Pluronic F127 and 0-10% Capryol 90.

**Figure S2** Influence of a temperature ramp on G’ (A), G” (B), and tan δ (C) of PLO gels prepared with various concentration of lecithin. Effect of the oscillation shear stress (D) and the influence of the frequency (E) on the elastic modulus (G’) of PLO gels prepared with various concentrations of lecithin at 37 °C. Plot of viscosities of PLO gels prepared with various concentrations of lecithin at 25 °C (F).

**Figure S3** In vitro release profiles of docetaxel (DTX) and cisplatin (CIS) from various formulations at pH 7.4 in phosphate-buffered saline containing 0.5% Tween 80. (A) DTX release profile. (B) CIS release profile. Each shown point is the mean $\pm$ SD (*n* = 3).
